# Molecular Modeling in Drug Design

**DOI:** 10.3390/molecules24020321

**Published:** 2019-01-17

**Authors:** Rebecca C. Wade, Outi M. H. Salo-Ahen

**Affiliations:** 1 Heidelberg Institute for Theoretical Studies (HITS), Schloss-Wolfsbrunnenweg 35, 69118 Heidelberg, Germany; 2 Zentrum für Molekulare Biologie der Universität Heidelberg (ZMBH), DKFZ-ZMBH Alliance, Im Neuenheimer Feld 282, and Interdisciplinary Center for Scientific Computing (IWR), Im Neuenheimer Feld 205, 69120 Heidelberg, Germany; 3 Pharmaceutical Sciences Laboratory and Structural Bioinformatics Laboratory, Faculty of Science and Engineering, Åbo Akademi University, Biocity, Tykistökatu 6 A, FI 20520 Turku, Finland

This Special Issue contains thirteen articles that provide a vivid snapshot of the state-of-the-art of molecular modeling in drug design, illustrating recent advances and critically discussing important challenges. The eight Original Research Articles, three Reviews, one Opinion, and one Perspective explore the application of computational methods, ranging from virtual screening and pharmacophore modelling through artificial intelligence and machine learning to molecular dynamics simulation and enhanced sampling to drug design against diverse targets, including protein-protein interfaces and membrane protein receptors. The challenges for predictive methods addressed include molecular flexibility, solvation properties, hydrogen-bonding, and ligand polarization.

Three of the Original Research Articles describe the application of enhanced molecular dynamics (MD) simulation methods to drug design problems. Cao et al. [1] investigated ligand recognition in the neuronal adenosine receptor type 2A (hA_2A_R). This G-protein coupled receptor (GPCR), a promising drug target for neurogenerative diseases, was embedded in a solvated neuronal-like membrane and its interaction with a high-affinity antagonist was studied by well-tempered metadynamics. These calculations were confirmed by experimental binding affinity studies and they suggest the importance of interactions between membrane lipids and the protein extracellular loops in the ligand recognition process. The results give valuable insight for the design of hA_2A_R ligands, as well as other GPCR targeting ligands. Kouza et al. [2] explore peptide-protein interactions using steered molecular dynamics (SMD) simulations. By calculating the mechanical stability of ligand-protein complexes, SMD gives an effective alternative to binding affinity for assessing the strength of the binding interactions. The authors tested a novel pulling direction along the resultant dipole moment (RDM) vector in probing the mechanical resistance of a peptide-receptor system and observed that it results in stronger forces than the commonly used pulling direction along the centre of masses vector. This observation could be utilized in improving the ranking of ligand binding affinities by using mechanical stability as an effective scoring function. A similar approach was taken by Tavanti, Pedone, and Menziani [3], who present a systematic computational study of the effect of natural biophenols on the destabilization of preformed amyloid-β(1-40) fibrils. They applied the replica exchange molecular dynamics (REMD) approach to identify the possible ligand binding sites on the fibrils, the molecular mechanics Poisson-Boltzmann surface area (MM-PBSA) method to calculate the binding free energies of the ligands at these binding sites, and then used an SMD-type approach to investigate how the ligands affected the fibril stability by calculating the forces for pulling apart a protofibril double-layer during MD simulations in the presence of ligand. Importantly, they found that the lateral aggregation of the fibrils is significantly affected by the intercalation of the ligands. This observation may assist in rational inhibitor design targeting amyloid-β-fibril formation in Alzheimer’s disease. In their Review, Defelipe et al. [4] discuss the potential of MD simulations of solvated proteins for identifying the binding modes and binding free energies of new drug candidates, with a particular focus on the application of MD simulations with mixed solvents (effectively enhanced solvents) to efficiently identify the putative drug binding sites.

Applications of virtual screening and molecular docking are described in three of the Original Research Articles. Chen et al. [5] carried out a virtual screening study using a ligand-based pharmacophore approach to identify potential squalene synthase (SQS) inhibitors from a Traditional Chinese Medicine database. Subsequent molecular docking and MD simulation studies led them to select cynarin as a potential SQS inhibitor. It was shown to have a lipid lowering effect in a cell model. As cynarin did not map with the pharmacophore models of other possible anti-hyperlipidemia targets that are present in these cells, it may exhibit this activity by inhibiting SQS. Viviani et al. [6] show in their study how computationally predicted aggregators that are found in a virtual screening campaign for inhibitors of human *ecto*-5-nucleotidase inhibitors were actually inhibiting the enzyme due to aggregate formation. Their study underlines the importance of not only filtering the virtual hits by predicting their aggregate forming potential computationally, but also of experimental assays for aggregation. The study by Vincenzi, Bednarska and Leśnikowski [7] highlights the current limitations of molecular docking programs. They developed a virtual screening protocol for adenosine derivatives that were substituted with either a boron cluster or a phenyl group. Since flexible ligand docking tools that have been parameterized for modelling hexa-coordinated boron are lacking, the authors tested a rigid-body docking tool, PatchDock, which uses simple geometric shape complementarity to identify the docking poses and rank the ligands. Despite the simplicity, the results from the radioligand assays of the synthesized highest/lowest scoring compounds at the adenosine A2A and A3 receptors were rather consistent with the *in silico* predictions.

Two of the Reviews discuss the application of a combination of molecular docking and MD simulation-based approaches for target-based drug design. Krammer and co-workers [8] review the design of non-antibiotic anti-adhesives against the bacterial adhesin FimH, emphasizing the significance of the incorporation of the dynamic aspects of ligand-target interactions in drug design studies. Likewise, Ferraro and Colombo [9], in their Perspective, show examples of how MD simulations, in concert with screening approaches, can help in tackling challenging protein–protein interactions and designing therapeutic small molecules that inhibit such interactions. Nevertheless, there is clearly a need for methodological improvements. In their Expert Opinion, Pantsar and Poso [10] take up many critical aspects of molecular docking, such as the accuracy of the current scoring functions, the role of water in the binding site, the limited description of hydrogen bonding interactions, as well as the neglect of the dynamics of the system. The authors give valuable insights and tips for tools that can help to overcome some of the challenging issues and improve the reliability of binding affinity predictions.

Two Original Research Articles address methodological advances. Jedwabny, Lodola, and Dyguda-Kazimierowicz [11] test an *ab initio*-quantum mechanics-based scoring model to rank the affinities of a set of lithocholic acid derivatives at the ligand-binding domain of the erythropoietin-producing hepatocellular carcinoma subtype 2 (EphA2) receptor. These inhibitors prevent the physiological ligand ephrin-A1 from binding to EphA2, thus showing potential for becoming leads for future anti-cancer agents. This simple scoring model, comprising long-range multipole electrostatic and approximate dispersion interactions, yielded comparable or better binding affinity predictions than any of the tested empirical scoring functions. On the other hand, Mortier, Dhakal, and Volkamer [12] have developed a novel tool, truly target focused (T^2^F) pharmacophore modelling, to identify pharmacophoric features at protein surfaces. These features represent the key favourable interaction possibilities of ligands binding to the particular site. Such a target-based pharmacophore model can be valuable in drug design cases where the target protein structure is available, but there is limited information about possible ligands binding to the target. In addition, the tool can be used for exploring allosteric pockets and protein-protein interactions for possible ligand sites. Lastly, in their Review, Hessler and Baringhaus [13] give an overview of the important role of artificial intelligence, and, in particular, novel algorithms based on neural networks, in drug design. They focus especially on recent advances in the areas of activity and property prediction, as well as *de novo* ligand design and retrosynthetic approaches. While machine learning has long been used for drug design, new methods and applications are currently appearing at a rapid pace and, together with contemporary molecular modelling and simulation approaches, can be expected to improve the quality and value of computational approaches to drug design. 

This special issue is accessible through the following link: https://www.mdpi.com/journal/molecules/special_issues/MMDD.
